# Cardiac Magnetic Resonance Findings and Their Association with Clinical Outcomes in Pediatric Pulmonary Arterial Hypertension: An Exploratory Study

**DOI:** 10.3390/jcm15031107

**Published:** 2026-01-30

**Authors:** Meryem Beyazal, Merter Keceli, Oguzhan Dogan, Ibrahim Ece

**Affiliations:** 1 Pediatric Cardiology Department, Children Hospital, Ankara Bilkent City Hospital, Ankara 06800, Turkey; oguzhandogan32@hotmail.com (O.D.); dribrahimece@gmail.com (I.E.); 2 Pediatric Radiology Department, Children Hospital, Ankara Bilkent City Hospital, Ankara 06800, Turkey; merterkeceli@gmail.com

**Keywords:** pediatric pulmonary arterial hypertension, cardiac magnetic resonance, right ventricular function, prognosis

## Abstract

**Background**: Cardiac magnetic resonance [CMR] is a non-invasive tool to assess ventricular function in pediatric pulmonary arterial hypertension [PAH]. However, CMR parameters in children remain underexplored. **Methods**: Thirty-six children with PAH were prospectively evaluated using CMR. Right and left ventricular volumetric and functional parameters, including right and left ventricular ejection fraction [RVEF, LVEF], right and left ventricular end-systolic volume indexed to body surface area [RVESVi, LVESVi], right ventricular mass index [RVMi], ventricular mass index [VMI], septal curvature duration index [SCDI], and regional area change [RAC], were assessed. Clinical variables included brain natriuretic peptide [BNP], New York Heart Association [NYHA] class, and six-minute walk distance [6MWD]. Correlations, logistic regression, and Kaplan–Meier analyses were performed to determine associated factors for mortality. **Results**: RVEF was negatively correlated with BNP [r = −0.538, *p* = 0.001], while no correlation was found with LVEF. Decreased RVEF and LVESVi and VMI were associated with mortality in univariate analysis. Patients with VMI > 0.75 or leftward septal shift had significantly lower one-year survival [*p* = 0.016 and *p* = 0.040, respectively]. SCDI and RAC were not associated with mortality. **Conclusions**: RVEF, LVESVi, and VMI are associated with mortality in pediatric PAH. BNP reflects right ventricular dysfunction. VMI and septal morphology are strong associated markers and may enhance risk stratification in children with PAH.

## 1. Introduction

Cardiac magnetic resonance [CMR] imaging is a part of comprehensive examinations in the clinical follow-up of patients with pulmonary hypertension [PH]. Right ventricular volumetric parameters such as right ventricular end-systolic volume [RVESV], right ventricular ejection fraction [RVEF], and right ventricular stroke volume [RVSV] were included in the European Society of Cardiology [ESC] risk classification [1]. Additionally, increased right ventricular end-diastolic volume [RVEDV], decreased left ventricular end-diastolic volume [LVEDV], and increased right atrial volume [RAV] have also been associated with prognosis [2,3]. In addition, some newly determined indices, such as ventricular mass index [VMI = right ventricular mass/left ventricular mass ratio], septal curvature duration index [SCDI = defined as the proportion of CMR frames with a septal bow toward the left that was present during one cardiac cycle], and regional area change [RAC], indicating pulmonary artery stiffness, can be used as prognostic parameters [2,4,5].

CMR-based prognostic studies in children with pulmonary hypertension are limited compared to adults. While pediatric studies have similarities to adult studies, differences have also been demonstrated. RVEF has prognostic significance in children as it does in adults [6], but some volumetric parameters such as RVSV, RVEDV, and LVEDV have been noted as not having prognostic significance in children [7]. In addition, regarding the left ventricular eccentricity index [the ratio of the anterior–inferior and septal–posterolateral cavity dimensions at the mid-ventricular level], pediatric studies are noteworthy. It correlates with outcomes in children with PAH and bronchopulmonary dysplasia [BPD]-associated PAH [8,9].

The distribution of etiologies in pediatric PH is quite different to that of adults, with children having a greater predominance of idiopathic pulmonary arterial hypertension [IPAH] and pulmonary arterial hypertension associated with congenital heart disease [PAH-CHD]. In newborns, pulmonary hypertension is primarily caused by bronchopulmonary dysplasia in premature infants and persistent pulmonary hypertension in term infants due to meconium aspiration, respiratory distress syndrome, and congenital diaphragmatic hernia [10]. In addition, intrauterine closure of ductus arteriosus may lead to persistent pulmonary hypertension [11].

In this study, we aimed to determine CMR-based associated factors and the relationship between CMR results and clinical parameters in patients diagnosed with childhood PAH.

## 2. Material and Methods

### 2.1. Study Design

This retrospective cross-sectional study was conducted with 36 consecutive patients who were diagnosed with PAH in our institution between 2021 and 2025 according to the following criteria. The hemodynamic definition of PH as applied to infants and children beyond the first months of life continues to be the same as in adults, defined as a mean pulmonary arterial pressure [mPAP] ≥ 20 mmHg at rest determined by cardiac catheterization. For the definition of pre-capillary PH [including pulmonary arterial hypertension and PH-associated lung disease] in infants and children, a pulmonary capillary wedge pressure < 15 mmHg and a pulmonary vascular resistance index [PVRI] > 3 Wood Units·m^2^ [WU·m^2^] is recommended [12].

**Figure 1:**
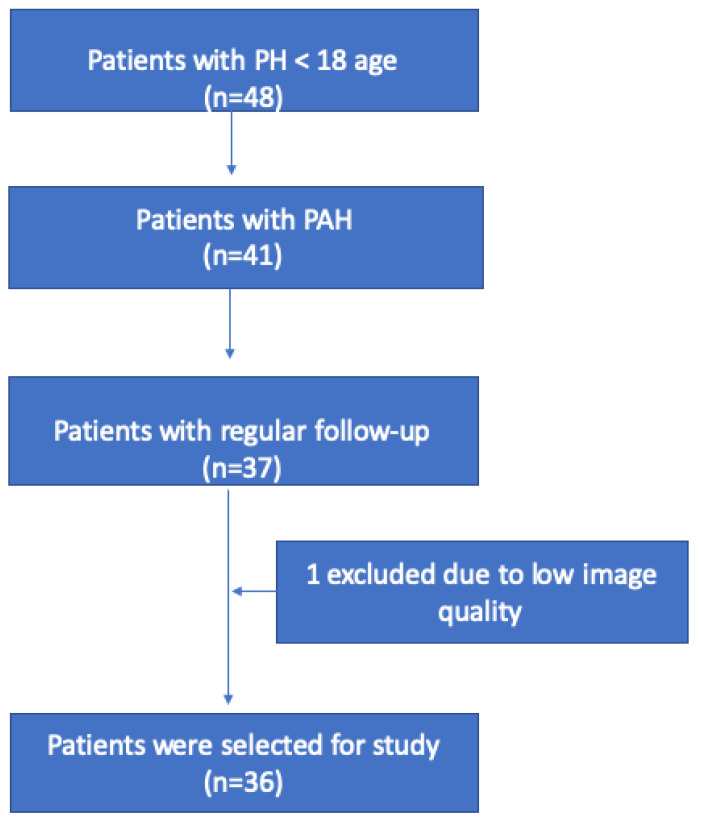
The flow chart of sample size (PAH = Pulmonary arterial hypertension, PH = Pulmonary hypertension).

Inclusion criteria include being under 18 years of age at the time of PAH diagnosis and having either idiopathic PAH or PAH associated with congenital heart disease. All patients with PAH-CHD had an untreated heart defect with right to left shunting. CMR was performed within one year of the diagnosis for all PAH patients. One patient was excluded from the study due to poor image quality [Figure 1].

### 2.2. Data Collection

The patients’ data was obtained from electronic medical records. Clinical and laboratory data included age at CMR, weight, height, body surface area [BSA], heart rate [HR], the type of anti-pulmonary hypertension [anti-PAH] medication and its usage period, single or multiple anti-PAH use status, six-minute walk distance [6MWD], pre–post oxygen saturation results in the six-minute walk test [6MWT], BNP level, and current NYHA class.

### 2.3. Cardiac Magnetic Resonance

The CMR protocol at our institution, which is routinely applied, is presented in Table 1. This protocol was created according to the Society of Cardiac Magnetic Resonance [SCMR] guidelines and the European Association of Cardiovascular Imaging [EACVI] textbook [13,14].

All CMR studies were performed using a 1.5-Tesla system [GE Healthcare, Chicago, IL, USA] in line with the above-mentioned protocol, and the cardiac-VX program [AW VolumeShare 7, Milwaukee, WI, USA] was used for post-processing. Data was acquired during breath holding or free breathing if the patients were sedated. Breath-hold steady-state free precession [SSFP] sequence parameters were as follows: repetition time [TR] 4.2 ms, echo time [TE] 2 ms, flip angle 60°, slice thickness 6 mm, in-plane image resolution 1.6 mm × 1.6 mm × 6.0 mm, and temporal resolution 69 ms. The sequence parameters during free breathing were as follows: TR 3.8 ms, TE 1.6 ms, flip angle 60°, slice thickness 5 mm, and in-plane image resolution 1.6 mm × 1.6 mm × 5.0 mm. Short axis cine images were used for ventricular volumetric analysis. The endocardial and epicardial borders of both ventricles were traced manually at end-systole and end-diastole. Then, the end-diastolic volume, end-systolic volume, stroke volume, ejection fractions of right ventricle and left ventricle, right and left ventricular mass, and cardiac output [CO] were calculated. All volumetric parameters were indexed to body surface area. Ventricular mass index was calculated by dividing right ventricular mass [RVM] by left ventricular mass [LVM]. Four-chamber cine images were used for right atrial volumes and indexed to BSA. In addition, the percentage of the entire cycle in which the septal shift is seen, known as the septal curvature duration index, was calculated by examining short axis slices.

Two-dimensional [2D] through-plane phase-contrast flow measurements were obtained from the ascending aorta [Aa], main pulmonary artery [MPA], and right and left pulmonary artery [RPA and LPA]. Flow measurements were performed perpendicular to each targeted vessel using the double oblique technique. In all cases, encoding velocity was adjusted to avoid aliasing. Initial velocity encoding [VENC] values were determined as 150 cm/s and 80–100 cm/s for the aorta and pulmonary arterial systems, respectively. The velocity encoding value is generally selected as small as possible for the pulmonary arteries, with a predefined upper velocity limit of 100 cm/s. In all cases, encoding velocity was adjusted to avoid aliasing. Imaging parameters that were used for the phase contrast imaging were as follows: TR/TE, 6.7 ms/4.2 ms; flip angle, 25°; phase to reconstructed 30; temporal resolution, 107 ms; the number of signal averages, 1; and section thickness, 7 mm. Field of view and pixel size [1.6–2.3 mm] were adjusted individually according to a patient’s size. Through 2D-phase contrast flow, Qp:Qs, mean velocity of pulmonary artery [mvPA], and pulmonary artery regurgitation fraction [PARF] were obtained. In addition, RAC determined the change in cross-sectional area during the cardiac cycle in a proximal portion of the pulmonary artery.

Respiratory navigator-gated, electrography-triggered, 3D whole-heart balanced SSFP magnetic resonance imaging was acquired during free breathing. Through this, PA and Ao diameter in diastole was measured. Lastly, after the injection of the gadolinium-based contrast agent [0.01 mmol/kg], late phase [10–20 min] images were obtained. Then, the presence of late gadolinium enhancement [LGE] and its localization were evaluated.

### 2.4. Statistical Analysis

All data were analyzed by using the SPSS, Statistical Package for Social Sciences for Windows 25.0 program [IBM Corp., Armonk, NY, USA]. After descriptive statistics and normality analysis, normally distributed continuous variables were reported as mean ± standard deviation, non-normally distributed continuous variables were reported as median with range, and categorical variables were reported as count with percentage of total. Correlation analysis was used to determine the relationship between two numerical variables. To determine the association of categorical or continuous independent variables with one dichotomous dependent variable, logistic regression analysis was used. The Kaplan–Meier method was used to estimate freedom from each of the specified endpoints, stratified by age, over the available follow-up time.

## 3. Results

The study included 36 pediatric patients with pulmonary arterial hypertension, whose baseline characteristics are summarized in Table 2. Of the patients, 24 [66.7%] were female, and the median age was 13.1 [6.4–17.6] years. The median follow-up was 14 [2–29] months. There was no significant difference in CMR findings between patients with different etiologies [Table 3].

**Figure 2:**
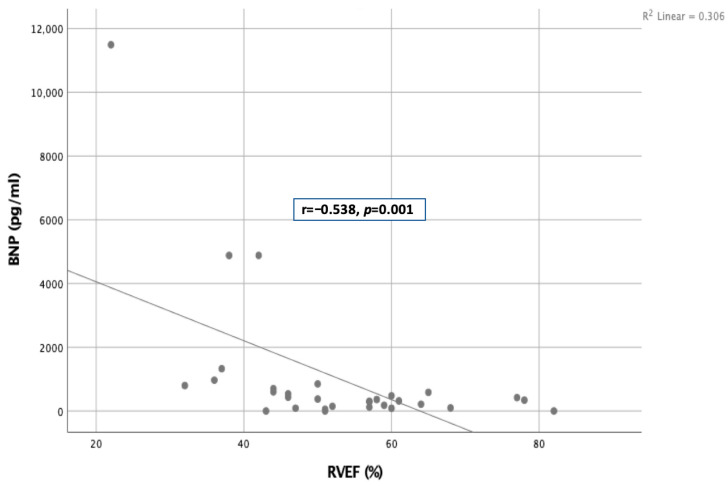
A negative moderate correlation between RVEF and BNP (RVEF = Right ventricular ejection fraction; BNP = Brain natriuretic peptide).

In the analysis of clinical parameters, a negative moderate relationship was found between RVEF and BNP [r = −0.538, *p* = 0.001] as shown in Figure 2. However, no significant relationship was found between BNP and left ventricular ejection fraction [LVEF]. In addition, there was no correlation between CMR volumetric parameters and other clinical variables such as the 6MWD and NYHA class. Additionally, RAC values were below 40% in all patients.

Mortality was observed in three of the patients [8.3%] during the follow-up. The univariate logistic regression analysis showed that decreased RVEF and left ventricular end-systolic volume index [LVESVi] increased VMI significantly and increased the risk of mortality. However, SCDI and RAC values were not found to be a risk factor for mortality. The logistic regression analysis results are given in Table 4.

**Figure 3:**
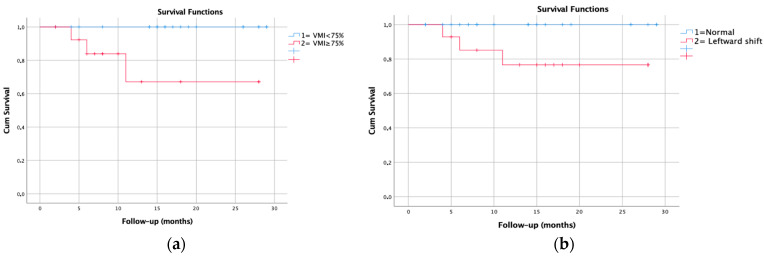
Kaplan–Meier mortality-free survival curve. (**a**) The transplant-free survival rate in patients with a VMI > 0.75 at the 1st year of follow-up. (**b**) The transplant-free survival rate with a presence of leftward septal shift at the first year of follow-up [VMI = Ventricular mass index].

According to the Kaplan–Meier survival analysis, in patients with a VMI > 0.75, there is a difference in mortality rates at approximately the fifth month of follow-up [*p* = 0.016, long rank = 5.827]. The transplant-free survival rate was found to be approximately 65% in patients with a VMI > 0.75 at the first year of follow-up [Figure 3a]. Similarly, in patients with leftward shift, there is a difference in mortality rates at approximately the fifth month of follow-up [*p* = 0.040, long rank = 3.860]. The transplant-free survival rate was found to be approximately 75% in patients with a presence of leftward septal shift at the first year of follow-up [Figure 3b].

## 4. Discussion

In this study, we discovered that among various clinical and laboratory tests, only BNP was associated with CMR parameters. Another finding was that increased VMI and decreased RVEF and LVESVi were associated with mortality. In those with a VMI > 0.75 and septal shift, mortality-free survival at the first year of follow-up was found to be approximately 65–75%.

BNP is an amino-peptide secreted by left ventricular and right ventricular myocytes as pre–pro-BNP [15]. Since the contribution of the right ventricle to hemodynamics is limited, BNP levels are well established markers for the prognosis and diagnosis of left ventricular disease. However, there are studies showing that pro-BNP is associated with right ventricular function and enlargement in patients with pulmonary embolism [16,17]. Similarly, Pasha SM et al. reported that, in comparing patients with pulmonary embolism with healthy controls, BNP was found to be associated with right ventricular functions in the patient group, while it was found to be associated with left ventricular functions in the healthy group [18]. Supporting this study, we found that BNP was negatively correlated with RVEF, without any correlation with LVEF, although the patient population was different. Our theory is that BNP may be associated with the ventricle, where functional impairment is evident.

The lack of a correlation between NYHA functional class and CMR volumetric parameters in this study may be attributed to the absence of NYHA class I patients. In the study by Shang X et al., RVEDV and RVESV showed a moderate and significant correlation when patients were grouped according to NYHA functional class (I/II vs. III) [19]. The most pronounced differences in functional status are likely to be observed in patients classified as NYHA class I. In addition, unlike the study by Lachant DJ et al. [20], we did not find an association between 6MWD and CMR parameters. Lachant et al. demonstrated a relationship between 6MWD and LVSVi in an adult population. The absence of such an association in our study may be explained by differences in age and underlying disease etiology.

A decrease in RVEF and an increase in RVESVi are well-established prognostic markers in patients with pulmonary hypertension according to adult studies [1,2,21]. Studies with children are limited. A pediatric study reported that RVEF was a prognostic factor, concurring with our results [6]. However, we did not find that RVSVi, which is included in the ESC risk stratification criteria, was associated with mortality. This result can be explained as follows: Given that right ventricular diastolic function decreases with age [22], the increase in RVEDVi may be more pronounced in children with PH compared to adults. This may have masked the prognostic value of RVSVi in children, since stroke volume will not decrease in case of a simultaneous increase in end-systolic and end-diastolic volumes.

The impact of decreased LVESVi on mortality in childhood PH may be due to altered left ventricular geometry. In a patient with right ventricular volume overload, an increase in left ventricular eccentricity index was found only at end-diastolic, whereas in a patient with pressure overload, both end-systolic and end-diastolic left ventricular eccentricity index were increased [23]. The increased eccentricity index seen throughout the cycle in PH patients is likely to be the result of volume changes in the ventricles [increased RVESVi and decreased LVESVi]. Although we were unable to find any publications on the prognostic impact of LVESVi for PH patients, according to Haarman’s study, the left ventricular eccentricity index was associated with transplant-free survival from the time of CMR [8]. This study highlights the importance of LVESVi.

Right ventricular hypertrophy and subsequent left ventricular hypertrophy secondary to increased pressure are common in patients with pulmonary hypertension. In the compensated phase of pulmonary hypertension, hypertrophy occurs in the right ventricle, while in the decompensated phase, hypertrophy of the left ventricle and dilatation of the right ventricle are observed [24]. The most important difference between the compensated and decompensated phases is that in the former, only the right ventricular myocardium is damaged, while in the latter, both ventricular myocardia are damaged. Septal bowing is also observed in the decompensated phase. In fact, this entire trajectory explains the seemingly contradictory studies showing how right ventricular hypertrophy affects prognosis. A study found that right ventricular mass > 59 g/m^2^ and VMI > 0.75 were independent predictors of mortality and adverse outcomes [2]. Another study reported that compensatory right ventricular hypertrophy was associated with better survival, while a decrease in right ventricular mass on serial examinations was a sign of poor prognosis [25]. While no hypertrophy is undoubtedly better than right ventricular hypertrophy, the decompensated phase, in which septal bowing begins and left ventricular hypertrophy and damage occur, is worse than the compensated phase. Therefore, the studies do not actually show any contradiction but rather indicate which phase the patient is in. We believe that the ventricular mass index, which recently has been used more frequently, is very valuable in understanding the mass in both ventricles and the ventricular pressure balance. Our study, supporting this general information, shows that VMI was associated with mortality in pediatric PAH patients. Furthermore, in survival analysis, we demonstrated that there was a difference in mortality curves after approximately five months between patients with and without a VMI greater than 0.75. In our opinion, in clinical practice, using VMI to assess left ventricular myocardial damage, rather than RVMi alone, would yield more valuable information.

Interventricular septal shift is an end-stage finding in PH that occurs after left ventricular myocardial damage [24], and it occurs when right ventricular pressure is ≥5 mmHg higher than left ventricular pressure [4]. According to a study, septal leftward shift has been related to mean pulmonary arterial pressure greater than 67 mmHg and was considered one of the strongest prognostic factors in PH, supporting our results [26]. Mouratoglou SA et al. also calculated the septal curvature duration index (defined as the proportion of CMR frames with a septal bow toward the left that was present during one cardiac cycle), and they found that it is associated with a worse prognosis if it lasts longer than 2/3 of the cardiac cycle [4]. However, our results do not support this study. According to our findings, although the presence of a septal shift significantly impacts survival, its duration was not found to be associated with mortality. The only difference between Mouratoglou SA et al. and our study is that our patient group was children. However, the lack of association between SCDI and mortality in pediatric PAH patients is difficult to explain. Therefore, studies with larger populations would be beneficial to shed light on this issue.

Regional area change is a sensitive test for identifying patients with mild PH and is used for early diagnosis. In addition, a study in adults reported that RAC was associated with a poor prognosis [27,28]. Stiffening of the proximal pulmonary artery occurs with age [29]. This may explain why we did not find RAC to be a risk factor in our study. According to our study, RAC can be used for early diagnosis in pediatric PH patients, but its relationship with mortality is unclear.

## 5. Limitations

This study has several limitations that should be acknowledged. First, it was conducted at a single tertiary center with a relatively small sample size, which may limit the generalizability of the findings and the statistical power, particularly for mortality analysis. Second, the follow-up period was relatively short, preventing assessment of long-term outcomes and dynamic changes in CMR parameters over time. Third, due to the limited number of events, multivariable regression analysis could not be reliably performed, and the results are therefore based on univariate analyses.

Additionally, CMR examinations were performed within one year of diagnosis rather than at uniform time points, which may have introduced variability related to disease progression or treatment response. Hemodynamic data from right heart catheterization were not contemporaneous with CMR in all patients, limiting direct correlation between imaging findings and invasive measurements. Finally, although advanced CMR indices such as SCDI and RAC were evaluated, their relationship with mortality in pediatric PAH may be influenced by age-related vascular and myocardial differences, which could not be fully accounted for in this study.

Despite these limitations, this study provides valuable insights into CMR-based risk stratification in pediatric pulmonary arterial hypertension and highlights parameters that warrant further investigation in larger, multicenter, longitudinal studies.

## 6. Conclusions

This study highlights the importance of CMR-derived parameters in children with pulmonary arterial hypertension. The negative correlation between BNP and RVEF, in the absence of a correlation with LVEF, may be explained by the preferential secretion of BNP from the more severely impaired ventricle. The lack of patients with NYHA class I may account for the absence of an observed relationship between NYHA functional class and CMR parameters. Additionally, unlike what was observed in adults, no correlation was found between 6MWD and CMR parameters; this may reflect age-related differences in functional capacity assessment.

Decreased RVEF and LVESVi and increased VMI were significantly associated with mortality. In particular, a VMI greater than 0.75 and the presence of septal leftward shift were strongly associated with poor outcome. These findings suggest that VMI and septal morphology reflect ventricular interaction and can be valuable markers for early risk stratification in pediatric PAH. Larger multicenter studies are needed to confirm these results.

## Figures and Tables

**Table 1:** CMR protocol applied in our institution for pulmonary hypertension.

Assessment	Sequence	Plane	Aim
Anatomy			
	Scout images	Axial, sagittal, and coronal	Morphology (hypertrophy, akinesia, hypokinesia, septal shift, the diameter of aorta and pulmonary arteries)
	Cine SSFP	2-C, 3-C, 4-C, LVOT, RV-2C, RV-3C, RVOT, RVOT cross-cut	
	3D-Whole heart	3D volume covering the heart and lung fields	
Function			
	SAX cine SSFP	A stack of short-axis slices from the base of the heart to the apex	EF, SV, EDV and ESV of the ventricle, LVCO, LV and RV mass
Flow	2D-phase contrast CMR	Aa, MPA, RPA, LPA	PARF, mvPA, Qp/Qs, RPA, and LPA flow distribution
Viability	LGE	SAX, 4-C	Scar

CMR = Cardiac magnetic resonance; SSFP = Steady-state free precession, C = Chamber; LVOT = Left ventricular outflow tract; RVOT = Right ventricular outflow tract; SAX = Short axis; EF = Ejection fraction; SV = Stroke volume; EDV = End-diastolic volume; ESV = End-systolic volume; CO = Cardiac output; 2D = Two dimensional; Aa= Ascending aorta; MPA = Main pulmonary artery; RPA = Right pulmonary artery; LPA = Left pulmonary artery; mvPA = Mean velocity of pulmonary artery; PARF = Pulmonary artery regurgitation fraction; LGE = Late gadolinium enhancement.

**Table 2:** Patients’ characteristics [*n* = 36].

Data *	
Gender	Female = 24 (66.7%) Male = 11 (30.6%)
Age (years)	13.1 (6.4–17.6)
Height (cm)	158 (101–175)
Weight (kg)	42 (24–87)
BSA (m^2^)	1.32 (0.9–2.0)
Follow-up (months)	14 (2–29)
Diagnosis	
IPAH	19 (52.7%)
PAH-CHD	16 (44.4%)
Porto-systemic shunt	1 (2.8%)
Medication use duration (months)	4.2 (2–18.4)
Anti-PAH	
Single	16 (44.4%)
Multiple	20 (55.5%)
NYHA class	
1	None
2	16 (44.4%)
3	14 (38.8)
4	6 (16.7)
Six-minute walking test	
Distance (m)	450 (150–650)
SpO2 before the test	94 (63–100)
SpO2 after the test	86 (40–98)
SpO2 differences	6.5 (0–31)
BNP (pg/mL)	360 (0–11,490)

* Data were presented as *n* (%) for categorical variables and median (minimum–maximum) for continuous variables BSA = Body surface area; IPAH = Idiopathic pulmonary arterial hypertension; PAH-CHD = Pulmonary arterial hypertension associated with congenital heart disease; PAH = Pulmonary arterial hypertension; NYHA = New York Heart Association; SpO2 = Blood oxygen saturation; BNP = Blood natriuretic peptide.

**Table 3:** CMR findings according to underlying etiology.

	IPAH (*n* = 19)	PAH Associated Heart Disease (*n* = 17)	*p* Value
RV			
EF (%)	52 (22–82)	54 (24–77)	0.585
EDVi (mL/m^2^)	88 (36–221)	86 (39–201)	0.562
ESVi (mL/m^2^)	41 (10–142)	41 (16–152)	0.765
SVi (mL/m^2^)	44 (18–79)	41 (23–75)	0.476
RVMi (gr)	42 (12–168)	39 (23–89)	0.890
LV			
EF (%)	63 (49–85)	58 (31–85)	0.196
EDVi (mL/m^2^)	56 (25–136)	61 (40–168)	0.253
ESVi (mL/m^2^)	17 (4–53)	28 (6–65)	0.169
SVi (mL/m^2^)	32 (21–83)	36 (15–103)	0.497
COI (mL/m^2^)	2.9 (1.3–7.1)	3.1 (2.4–13.9)	0.086
LVMi (gr)	49 (30–90)	56 (21–102)	0.227
VMI	0.74 (0.19–4)		0.221
RV/LV EDVi	1.82 (0.71–4.59)	1.10 (0.56–3.65)	0.175
RV/LV ESVi	2.31 (0.53–19.17)	1.25 (0.70–13.8)	0.233
RAVi (mL/m^2^)	37 (10–111)	28 (14–66)	0.235
Flow			
mvPA (cm/s))	85 (44–140)	85 (49–162)	0.930
PARF (%)	2 (0–10)	1 (0–10)	0.642
Qp: Qs	0.7 (0.4–0.8)	0.8 (0.3–0.9)	0.413
PA/Ao ratio	1.5 (0.8–3.2)	1.4 (0.9–2.8)	0.726
SCDI	43 (0–80)	38 (0–90)	0.905
RAC (%)	22 (7–34)	11 (4–33)	0.177

Significant difference (*p* < 0.05) CMR = Cardiac magnetic resonance; IPAH = Idiopathic pulmonary hypertension; PAH = Pulmonary arterial hypertension; RV = Right ventricle, LV = Left ventricle; EF = Ejection fraction; EDVi = End-diastolic volume index; ESVi = End-systolic volume index; SVi = Stroke volume index; RVMi = Right ventricular mass index; LVMi = Left ventricular mass index; COI = Cardiac output index; VMI = Ventricular mass index; RAVi = Right atrial volume index; mvPA = mean velocity of pulmonary artery; PARF = Pulmonary artery regurgitation fraction; PA = Pulmonary artery; Ao = Aorta; SCDI = Septal curvature duration index; RAC = Regional area change.

**Table 4:** The univariate regression analysis for mortality based on CMR results.

	Univariate Analysis	
	OR (95% CI)	*p* Value
RV		
EF (%)	0.87 (0.76–0.99)	0.045
EDVi (mL/m^2^)	1.02 (1.00–1.05)	0.054
ESVi (mL/m^2^)	1.03 (1.00–1.06)	0.036
SVi (mL/m^2^)	0.98 (0.91–1.05)	0.587
RVMi (gr)	1.06 (1.00–1.12)	0.043
LV		
EF (%)	1.20 (1.03–1.39)	0.089
EDVi (mL/m^2^)	0.92 (0.83–1.02)	0.148
ESVi (mL/m^2^)	0.75 (0.57–0.98)	0.040
SVi (mL/m^2^)	1.01 (0.96–1.06)	0.669
COi (L/min/m^2^)	1.09 (0.75–1.61)	0.628
LVMi (gr)	0.98 (0.92–1.04)	0.527
Ratios		
VMI (RVMi/LVMi)	3.55 (1.22–10.57)	0.020
RV/LV EDVi ratio	3.92 (1.45–13.47)	0.065
RV/LV ESVi ratio	1.57 (1.03–2.40)	0.089
RAVi (mL/m^2^)	1.01 (0.97–1.05)	0.478
Flow study		
mvPA (cm/s))	0.96 (0.90–1.02)	0.187
PARF (%)	1.18 (0.85–1.64)	0.307
Qp:Qs	0.01 (0.0001–1.91)	0.089
Tissue characterization (LGE)		
RV-LV insertion (basal)	0.29 (0.04–1.84	0.191
RV-LV insertion (mid-ventricular)	0.58 (0.09–3.68)	0.566
Septal extending	0.61 (0.05–7.12)	0.700
PA/Ao ratio	0.79 (0.07–8.21)	0.850
SCDI	1.01 (0.97–1.04)	0.535
RAC (%)	1.05 (0.92–1.21)	0.412

Significant difference (*p* < 0.05) CMR = Cardiac magnetic resonance; OR = Odds ratio; RV = Right ventricle, LV = Left ventricle; EF = Ejection fraction; EDVi = End-diastolic volume index; ESVi = End-systolic volume index; SVi = Stroke volume index; RVMi = Right ventricular mass index; LVMi = Left ventricular mass index; COI = Cardiac output index; VMI = Ventricular mass index; RAVi = Right atrial volume index; mvPA = Mean velocity of pulmonary artery; PARF = Pulmonary artery regurgitation fraction; PA = Pulmonary artery; Ao = Aorta; LGE = Late gadolinium enhancement; SCDI = Septal curvature duration index; RAC = Regional area change.

## Data Availability

No new data were created or analyzed in this study.
